# A bibliometric analysis of research progress on pharmacovigilance and cancer from 2002 to 2021

**DOI:** 10.3389/fonc.2023.1078254

**Published:** 2023-01-25

**Authors:** Rina Sa, Yi Xu, Xinbo Pan, Yu Wang, Zhijian Lin, Xiaomeng Zhang, Bing Zhang

**Affiliations:** ^1^ School of Chinese Materia Medica, Beijing University of Chinese Medicine, Beijing, China; ^2^ Department of Pharmacy, Gansu Provincial Hospital, Lanzhou, Gansu, China; ^3^ Center for Pharmacovigilance and Rational Use of Chinese Medicine, Beijing University of Chinese Medicine, Beijing, China; ^4^ Institute of liver diseases, The Second People’s Hospital of Lanzhou, Lanzhou, China

**Keywords:** pharmacovigilance, cancer, bibliometric analysis, visualized analysis, research frontiers

## Abstract

The complexity of cancer itself and treatment makes pharmacovigilance critical in oncology. Despite rapid progress on pharmacovigilance and cancer research in the past two decades, there has been no bibliometric analysis in this field. Therefore, based on the Web of Science database, we used CiteSpace, VOS-viewer and R-bibliometrix to analyze and visualize publications, and described the development trend and research hot spots in this field. 502 publications were included. The development of pharmacovigilance and cancer research has continued to grow. The USA has the largest number of publications and citations, followed by France and UK. Vanderbilt University and Sorbonne University are the institutions that contribute the most papers, and 5 of the top 10 high-yield institutions are from France. Salem JE and Lebrun-Vignes B of Sorbonne University have published the most papers, and they have a strong cooperative relationship. Salem JE has the highest H index. *Drug Safety* has the largest number of publications in the field of pharmacovigilance and cancer, with a high impact factor (IF). In recent years, immune checkpoint inhibitors (ICIs) have been identified as a hot topic and will continue to be maintained. This paper can help researchers get familiar with the current situation and trend of pharmacovigilance and cancer research, and provide valuable reference for the selection of future research directions.

## Introduction

Cancer is a significant cause of death worldwide. According to *the Global Cancer report 2020* published by the International Agency for Research on Cancer in 2021(https://www.iarc.who.int/), there are about 19.3 million new cases of cancer, and 10 million cancer deaths worldwide in 2020. It was estimated that there will be 28.4 million new cancer cases worldwide by 2040 (1). The urgent need to control tumor development and prolong life has accelerated the rapid development of treatment methods. Whether traditional cytotoxic, hormonal, small molecule targeted agents or emerging antibody classes, antibody conjugated agents, ICIs are effective in killing cancer cells, improving patient quality of life, and prolonging survival (2–6). However, inevitable adverse drug reactions (ADRs), drug resistance, or relatively inadequate clinical experience limit their use and may exacerbate cancer progression and complicate treatment. For example, anthracyclines are effective agents for the basic treatment of many solid tumors and hematologic lymphatic system malignancies. However, some serious toxic effects seriously affect the clinical effectiveness, including bone marrow toxicity and cardiotoxicity (7–9). Even the cutting-edge innovations, such as anti programmed cell death protein 1 (PD-1), anti programmed cell death protein (PD-L1) or anti cytotoxic T-lymphocyte-associated protein 4 (CTLA-4), while greatly improving tumor killing rates and prolonging survival period of patients, their unique immune adverse events such as skin toxicity, gastrointestinal toxicity, pneumonia and endocrine toxicity also limit their use (10, 11). Therefore, the adverse effects of anti-neoplastic drugs are one of the most important problems tumor patients face during treatment. Timely and effective sorting and evaluation of the safety of anti-neoplastic drugs is necessary for rational clinical application.

Pharmacovigilance was defined by the World Health Organization (WHO) as science and activities relating to the detection, assessment, understanding and prevention of adverse effects or any other possible drug-related problems (12). Pharmacovigilance runs through the whole process of drug development and use, and its contribution is to recognize and quickly intervene the treatment-related risks, thereby improving the safety of drugs. For example, dose-dependent, cumulative and progressive cardiotoxicity is a serious ADRs of anthracyclines. Therefore, reducing the cumulative dose of anthracyclines can reduce heart failure-related complications (13). Compared with traditional systemic chemotherapy, the adverse reactions caused by the new targeted therapy are more rapid and acute because they involve autoimmune reactions and unpredictable inflammatory or allergic reactions (14, 15). In addition, the latest evidence shows that ICIs also have the risk of long-term toxicity, which can lead to endocrine diseases (such as hypothyroidism or type I diabetes) and rheumatism (such as arthritis), which will greatly affect the survival period and quality of life of patients (16). So the patients taking these drugs must be monitored closely to identify new ADRs. Spontaneous reporting systems are the most common and direct form of pharmacovigilance, providing early warning and control of safety issues related to marketed drugs. However, spontaneous reporting systems often have limitations, such as under-reporting, late reporting, erratic reporting rates, causality difficult to determine and so on (17, 18). Of these, under-reporting is the major limitation, which may be related to health professionals’ perceptions (belief that only serious ADRs need to be reported or uncertainty about whether a drug is the cause of a particular ADRs), fear of hassle (complicated reporting process), and limited resources (lack of IT tools in some regions). Therfore, proactive monitoring by health professionals can effectively reduce the incidence of ADRs and under-reporting (19). In addition, organizing regular training and reporting reminders to the patient information systems can also identify ADRs timely. In conclusion, considering the specificity of cancer itself, the complexity of treatment, and the continuous emergence of new therapeutic agents, it is necessary to strengthen pharmacovigilance in oncology in many ways, which is essential for the continued safety and effectiveness of anti-neoplastic drugs.

Bibliometrics is a science that uses mathematical and statistical methods to qualitatively and quantitatively evaluate published literature and visualize the data (20). It can analyze the distribution of countries/regions, authors and journals in the research, and help scholars quickly grasp the research hot spots and development trends in specific fields (21, 22). In a word, it provides objective scientific indicators for evaluating research achievements. Hundreds of papers on pharmacovigilance and cancer have been published, showing researchers’ continuous interest in this field. However, the bibliometric analysis of pharmacovigilance and cancer has not been reported yet. Therefore, this study is the first to use bibliometric methods to assess the status and trend of pharmacovigilance and cancer research, find research hot spots in this field, and provide valuable reference for further research.

## Materials and methods

### Data source and search strategy

Data from publications related to pharmacovigilance and cancer come from the Web of Science Core Collection (WoSCC). As one of the most authoritative and influential databases, WoSCC has comprehensive information and covers 18000 journals and 256 discipline categories (23, 24). It is essential for the collection and comparison of historical articles. The literature search was conducted on August 24, 2022, using a search strategy to search for initial data on pharmacovigilance and cancer by topic. The search terms were derived from the literature and Medical Subject Headings (MESH, https://www.ncbi.nlm.nih.gov/mesh). The search strategy was presented as follows: TS=((Tumor* OR Tumors* OR Cancer OR Cancers OR Neoplasm OR Neoplasia* OR Neoplasias OR Malignancy OR Malignancies OR Malignant Neoplasm OR Malignant Neoplasms OR Neoplasm, Malignant OR Neoplasms, Malignant OR Benign Neoplasms OR Benign Neoplasm OR Neoplasms, Benign OR Neoplasm, Benign)AND(Pharmacovigilance)).

**Figure 1:**
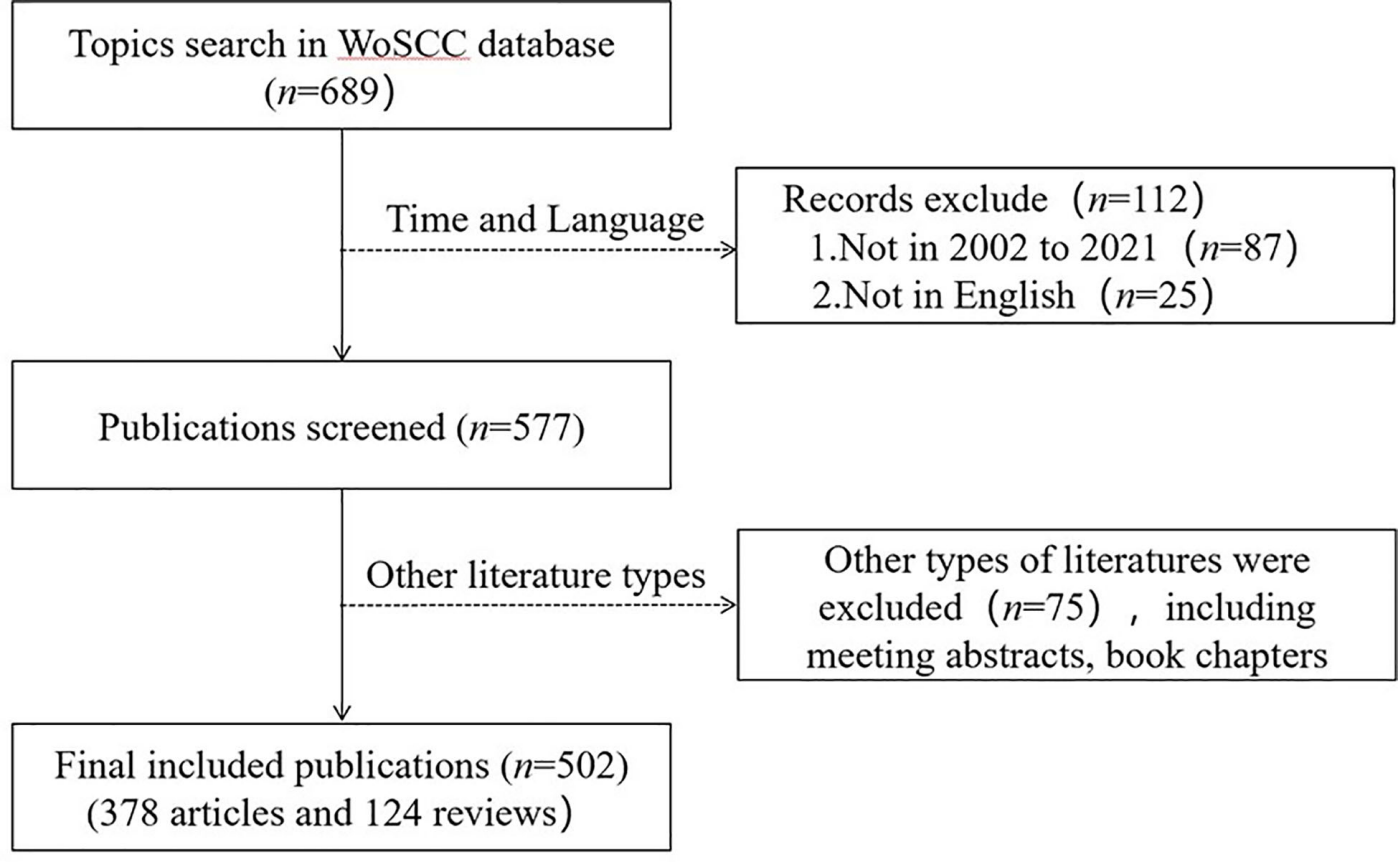
Flow chart of publication retrieval and selection.

Considering that the definition of Pharmacovigilance was formally improved by WHO in 2002, data were retrieved from 2002 even if no related articles were published in 2002, in order to determine the trend of obvious change and the value of analysis.The time span covers 20 years from January 1, 2002 to December 31, 2021. The publication language is set to English. The type of publication is article or review. **Figure 1** showns the specific retrieval strategy. A total 502 publications were collected from WoSCC for further analysis.

### Bibliometric analysis

We used CiteSpace, VOS-viewer, and R-bibliometrix to analyze literature and visualize bibliometric data, and Microsoft Excel (Office 2019) for data management.

CiteSpace (Version 5.8. R3) is a bibliometric analysis visualization tool based on the Java platform, which can be used for collaborative network analysis, co-occurrence and co-citation analysis, and then generate visual maps (25, 26). CiteSpace was used for keyword burst analysis and citation analysis. VOS-viewer (version 1.6.17) was applied to analyze the partnerships of countries/regions, institutions and authors (27). R-bibliometrix (version R 4.1.2) was utilized to analyze the research hot spots and trends with keywords, trend topics and thematic map. Moreover, researchers’ scientific output and influence can be quantified by the H index. Based on the 2021 Journal Citation Reports, impact factors (IF) were calculated for Journal.

## Results

### Annual output trend of publications

**Figure 2:**
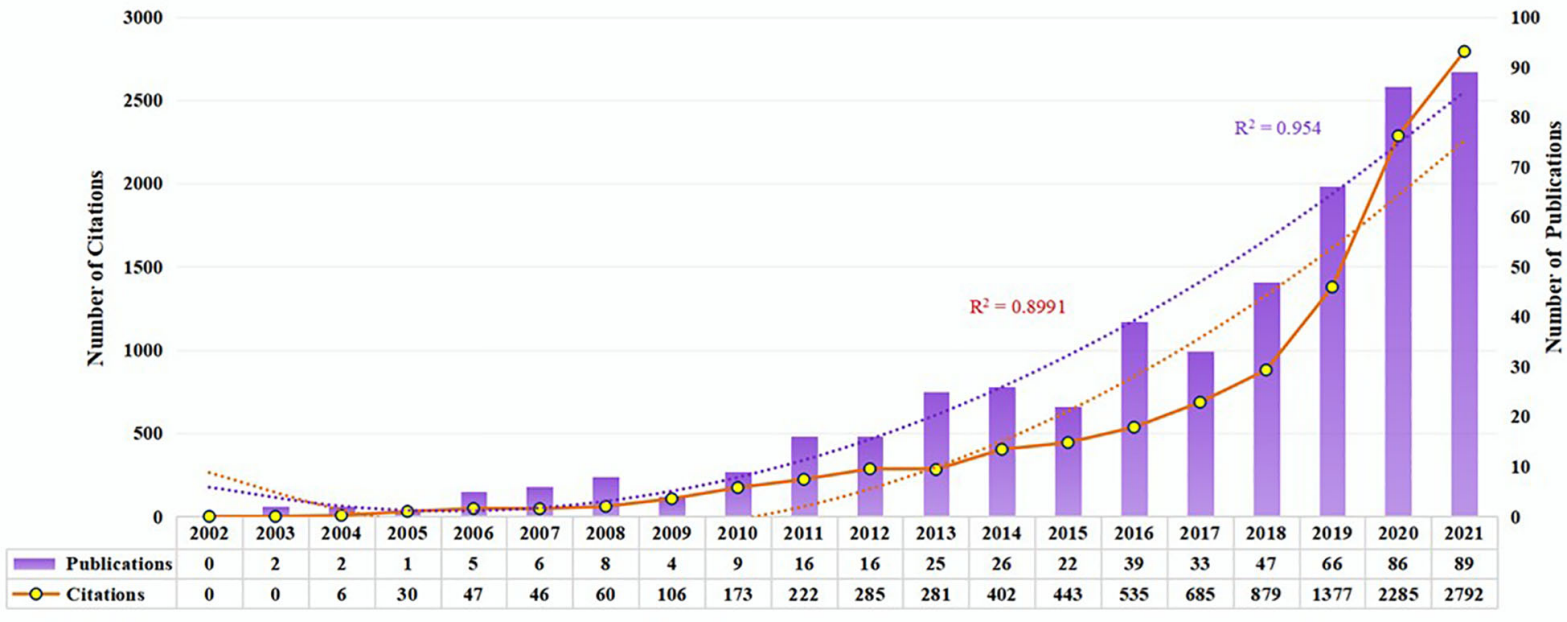
Trends in publications and citations from 2002 to 2021. The purple bars represent the number of publications per year. White nodes indicate the number of citations. Purple and orange dashed lines represent the growth curves of publications and citations, respectively.

Research development can be reflected in the number of publications. There were 502 publications obtained, including 378 articles and 124 reviews. According to **Figure 2**, the number of publications on pharmacovigilance and cancer is increasing annually. From 2002 to 2010, the output of publications was low, which meant that the research was at a standstill. The number of publications increased steadily from 2011 up to 2017, indicating that research in pharmacovigilance and cancer has begun to receive attention. From 2018 to 2020, the number of publications increased rapidly, with an annual growth rate of 30%~42%. By 2021, the growth rate of the number of publications had been decreased. There are 89 publications, close to the output in 2020. From the number of citations in publications, 507 papers were cited 10654 times, with an average of 21.01 per paper, and the number of citations increased steadily with time. The number of publications and citations on pharmacovigilance and cancer is on the rise, indicating that more researchers are attracted to this field.

### Cooperation and distribution of countries and regions

**Figure 3:**
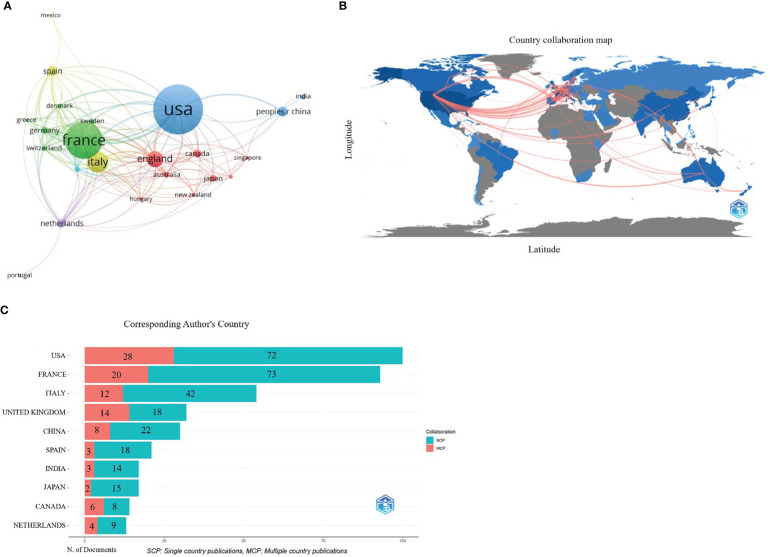
Countries/regions co-authorship analysis. **(A)** Cluster diagram of countries/regions (over 5 occurrences). Publication visualization map of 23 countries, forming 5 cooperation clusters (nodes with the same color). Node size represents the number of publications. Clusters with the same color represent closer cooperation. The lines represent the country’s collaboration. **(B)** Geographic maps in publication and collaboration of countries/regions. The blue color indicates the output rate of publications, the darker the blue color indicates more publications, and the gray color indicates no publications. The red line represents the cooperation between countries/regions. The thicker the line, the stronger the cooperation. **(C)** Histogram of cooperation in the top 10 productive countries/regions. The x-axis represents publication production, the y-axis represents countries. The red section represents multiple country/region collaborative publications (MCP), and the green section defines single country/region publications (SCP). The numbers in the bar chart represent the number of publications.

There were 502 publications from 68 countries/regions. **Table 1** shows the distribution of the top 10 countries/regions; Europe contains six, and the others are in North America, Asia, and Africa. Among them, the USA contributed the most publications (*n*=156, 31.08%), followed by France (*n*=116, 23.11%) and Italy (*n*=66, 13.15%), which accounted for more than half of all publications from 2002 to 2021. Besides, publications from the USA owned the highest citations (*n*=6738), followed by those from France (*n*=3863) and England (*n*=1742). The cluster analysis in **Figure 3A** shows more than five publications in 23 countries/regions. These results indicate that Europe and USA are leading the way in pharmacovigilance and cancer research. In addition, **Figure 3B** shows cooperation between different countries/regions. Darker blue indicates more publications, thicker red indicates stronger collaboration across countries/regions. In addition, the bar chart in **Figure 3C** clearly shows the three countries with the most international collaborations: the USA, France and UK. This phenomenon indicates that international collaboration in pharmacovigilance and cancer research is the future trend.

**Table 1:** Top 10 countries/regions with the most publications in pharmacovigilance and cancer.

Rank	Countries/regions	Publications	Rate(N/502) %	Citations
1	USA (North America)	156	31.076	6738
2	France (Europe)	116	23.108	3863
3	Italy (Europe)	66	13.147	1057
4	England (Europe)	51	10.159	1742
5	China (Asia)	31	6.175	259
6	Spain (Europe)	30	5.976	492
7	Netherlands (Europe)	27	5.378	937
8	Canada (North America)	25	4.980	479
9	Germany (Europe)	22	4.382	1434
10	Japan (Asia)	21	4.183	345

### Institutional contributions

**Figure 4:**
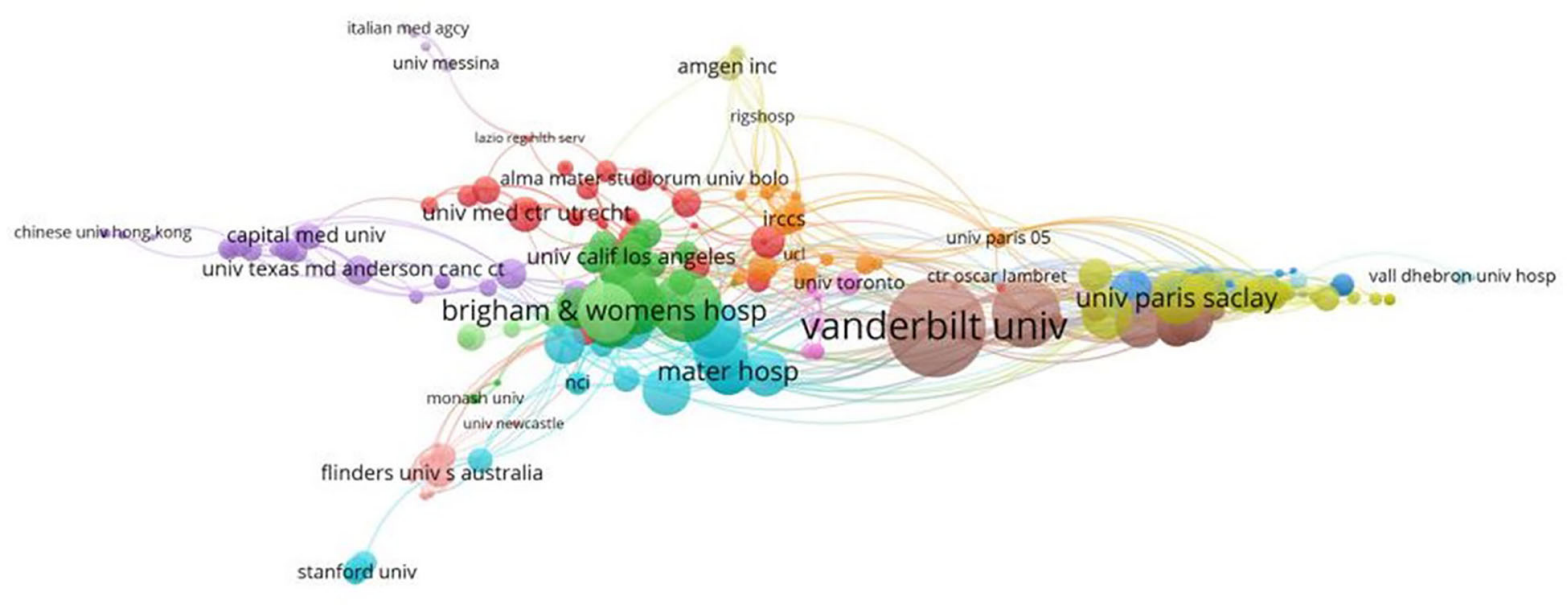
Cluster maps of the institutions. Nodes represent institutions. Node size is proportional to the contribution of the institution. Clusters of the same color imply more active cooperation. The line between nodes represents cooperation between institutions.

A variety of 1,328 institutions participated in the study. Five of the top 10 institutions are from France, and the others are from the USA, UK, Italy, and Canada. This suggests that France, the first country to introduce the concept of pharmacovigilance, has maintained pharmacovigilance research. As shown in **Table 2**, Vanderbilt University (*n* = 22, 4.38%) and Sorbonne University (*n* = 22, 4.38%) were also contributed the most papers, followed by the University of Bordeaux (*n* = 12, 2.39%). **Figure 4** shows the co-authorship network of 298 institutions, visualizing the two recorded thresholds. The institutions symbolized by the same color cooperated more actively.

**Table 2:** Top 10 institutions with a high volume of publications in pharmacovigilance and cancer research.

Rank	Institutions	Countries	Publications	Rate (N/502) %
1	Vanderbilt University	USA	22	4.38
2	Sorbonne University	France	22	4.38
3	University of Bordeaux	France	12	2.39
4	University of Toulouse	France	11	2.19
5	Northwestern University	USA	10	1.99
6	University of Manchester	UK	10	1.99
7	French National Institute of Health and Medical Research	France	9	1.79
8	University of Toronto	Canada	9	1.79
9	Istituto di Ricovero e Cura a Carattere Scientifico AVIANO CRO	Italy	8	1.59
10	University Of Paris-Sud 11	France	8	1.59

### Contribution of authors

**Figure 5:**
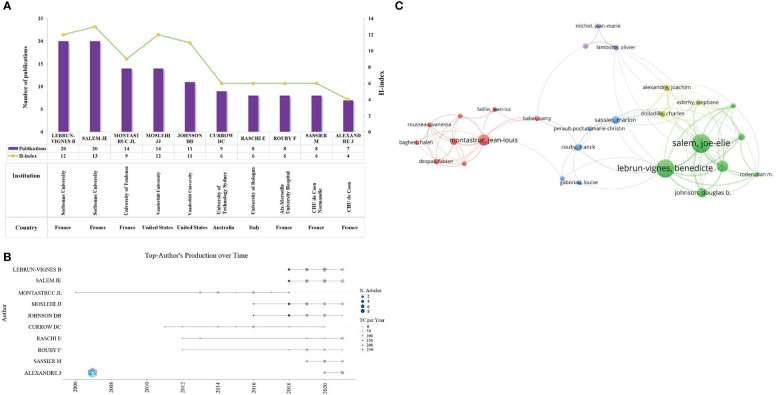
Co-authorship analysis. **(A)** The top ten authors with a high volume of publications and their H-index. The purple bars indicate the publications count, while the yellow nodes represent the H-index. **(B)** Annual production of the top 10 authors. The x-axis and y-axis represent the year and author, respectively. The larger the node, the more publications, and the darker the node’s color, the higher citations. **(C)** Cluster diagram of authors with more than 5 publications. The size of a node indicates the number of publications of an author. Clusters of the same color represent more active collaboration. Line thickness and distance between nodes indicate the relative strength of the relationship.

3150 researchers contributed to 502 publications on pharmacovigilance and cancer from 2003 to 2021. As shown in **Figure 5A**, the top 10 productive authors have 119 publications, accounting for 23.71% of all articles. Lebrun-Vignes B and Salem JE from Sorbonne University contributed the most papers (*n*=20, 20), followed by Montastruc JL from the University of Toulouse (*n*=14) and Moslehi JJ of Vanderbilt University (*n*=14). Salem JE from Sorbonne University owned the highest H-index (13), followed by Lebrun-Vignes B from Sorbonne University (12) and Moslehi JJ from Vanderbilt University (12). Which implies that these authors have a significant influence on the field of pharmacovigilance and cancer. **Figure 5B** shows a more detailed picture of the articles published by each author over time. Nodes with a darker color indicate more publications. It can be seen that Montastruc JL has been working in this field for the longest time; Lebrun-Vignes B and Salem JE have been concentrating on this field since 2018 and have a high number of academic outputs with high-quality outputs. **Figure 5C** shows a co-authorship network of 35 authors with more than 5 publications, demonstrating the extensive collaboration between different groups in the field of pharmacovigilance and cancer. This suggests direct or indirect collaboration between leaders in this research area.

### Analysis of academic journals

**Figure 6:**
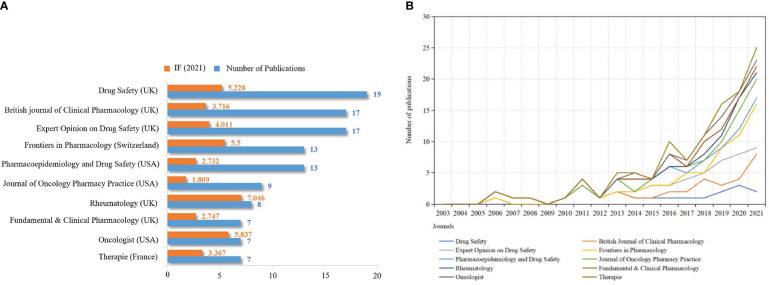
Quantitative analysis of journal publications. **(A)** Top 10 scholarly journals in publications and IF values. The orange and blue bars represent the IF value and the number of publications, respectively. **(B)** Annual publication output trend of the top 10 academic journals. X-axis: year; Y-axis: number of publications.

VOS-viewer software was used to analyze published journals visually. 261 academic journals published 502 publications. **Figure 6A** shows the top 10 academic journals with the most publications, half of which are from the UK. *Drug Safety* published the most papers (*n*=19), followed by the *British Journal of Clinical Pharmacology* (*n*=17) and *Expert Opinion on Drug Safety* (*n*=17). *Rheumatology* owned the highest IF (IF_2021 =_ 7.046), followed by *Oncologist* (IF_2021 =_ 5.837) and *Drug Safety* (IF_2021 =_ 5.228). This indicates that the above journals are of great academic influence in pharmacovigilance and cancer research. In addition, among 261 academic journals, about 27 journals (IF 0.701-35.855) belong to specific pharmacovigilance journals, such as *Drug Safety*, *Expert Opinion on Drug Safety* and *Pharmacoepidemiology and Drug Safety*, and about 19% of the articles published in these journals. This suggests that we should also focus on publishing articles in specific pharmacovigilance journals in the future, which is helpful to receive research advances timely in the field of pharmacovigilance. **Figure 6B** shows that the output of publications on pharmacovigilance and cancer in the top 10 journals has been growing in the past 19 years. The research of pharmacovigilance and cancer has attracted the attention of journals specializing in drug safety. In addition, research in pharmacoepidemiology, clinical pharmacology and therapy has also increased.

### Analysis of research category and funding agencies

**Figure 7:**
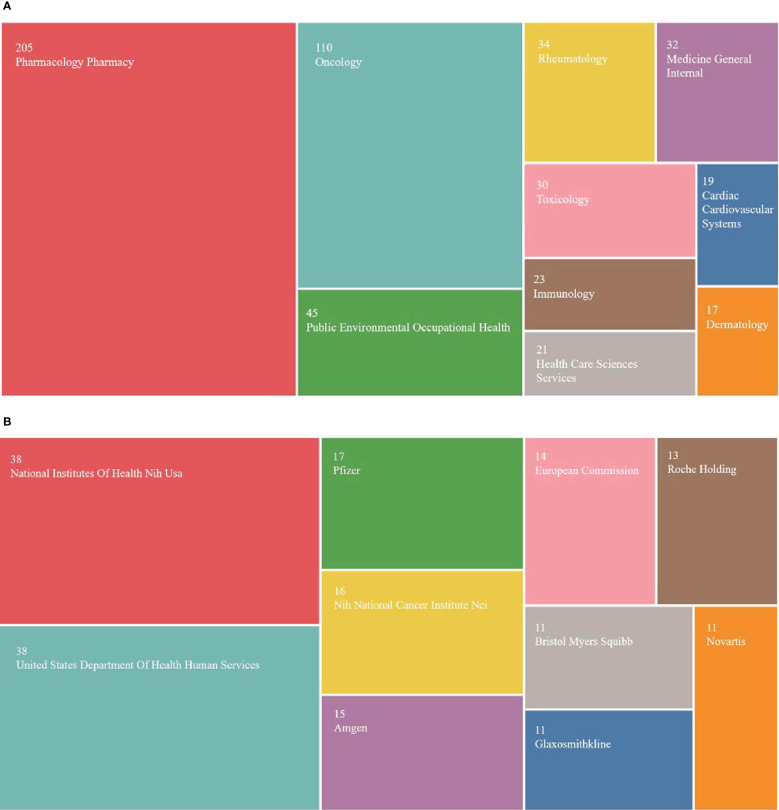
Distribution of research classifications and funding institutions. **(A)** Top 10 research classifications. **(B)** Top 10 funding institutions.

A total of 64 categories were involved in pharmacovigilance and cancer research. **Figure 7A** shows s the top 10 research classifications in this field. Pharmacology and pharmacy appeared most frequently (n=205), followed by oncology (n=110) and public environmental and occupational health(n=45). 425 of the 502 publications obtained funding support. The funding support rate was 84.6%, indicating that pharmacovigilance of antineoplastic drugs has gained attention globally. **Figure 7B** shows that five of the top 10 funding agencies are the National Institutes of Health (NIH) and the U.S. Department of Health and Human Services (HHS) (n=38, 38). The Nih National Cancer Institute Nic is also part of the NIH (n=16). This phenomenon suggests that USA has significantly contributed to pharmacovigilance and cancer research, which may be closely related to the country’s economic power and scientific strength. It also prompted us to explore the scope of research covered by the sponsored publications.

### Detection and analysis of keywords

**Figure 8:**
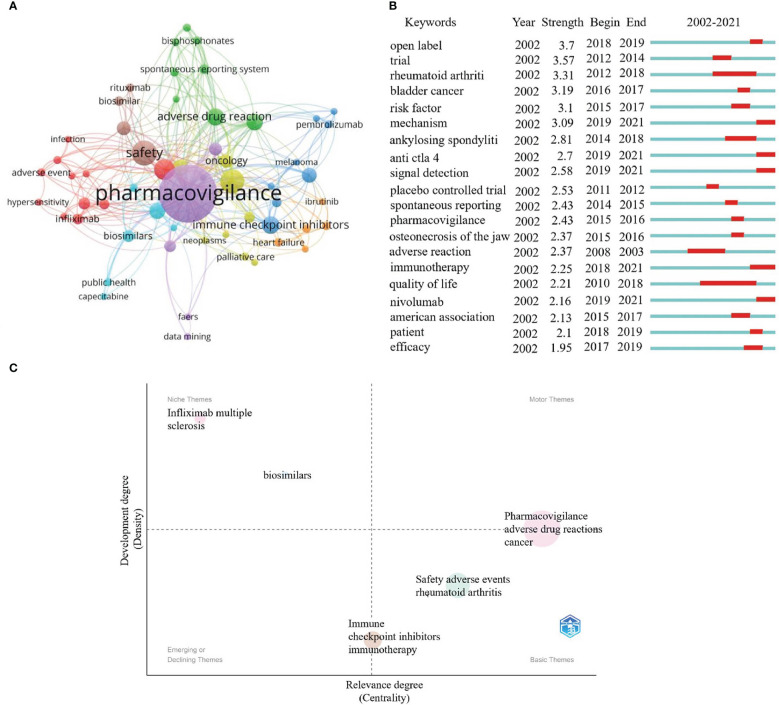
Keywords and thematic maps analysis. **(A)** Cluster profiling of keywords with a threshold of more than 5 frequencies. Nodes represent keywords. The node’s size is proportional to the frequency of keyword occurrence. Lines represents the connection between nodes. **(B)** Burst analysis of the previous 20 keywords. The blue line indicates the timeline from 2002 to 2021, whereas the red line represents the keyword burst period. **(C)** Thematic maps about the pharmacovigilance and cancer. The colored bubbles represent relevant keywords. The bubbles' size is positively proportional to the related keywords' frequency. The x-axis indicates the center and is used to measure the relevance between topics. y-axis represents the strength of the bearing, also known as density. The higher the density, the more mature the case.

Keywords are the centerpiece of a publication. Analyzing keywords can help us grasp the current status of research and explore the hotspots and directions in particular fields (20). There are 1189 keywords involved in this research. **Figure 8A** shows the top 10 high-frequency keywords as follows.: pharmacovigilance (158), safety (28), adverse drug reactions (29), cancer (30), immune checkpoint inhibitors (22), adverse drug reaction (21), drug safety (20), pharmacoepidemiology (20), adverse events (19), and immunotherapy (17). It indicates that these keywords are the research focus of pharmacovigilance and cancer.

These keywords can be divided into eight clusters with different colors. Purple indicates terms related to pharmacovigilance. Terms concerning evaluated indexes were in red, including adverse event, infection and hypersensitivity. The terms related to cancer immunotherapy research were blue and mainly included ICIs, melanoma, and pembrolizumab. Terms correlated with clinical studies were in yellow, mainly including oncology, neoplasms, and palliative care. Terms related to evaluate indexes of treatment were in green, primarily involving adverse drug reaction, spontaneous reporting system, and bisphosphonates. Terms concerning evaluated indexes of pharmacovigilance and cancer were in orange red, mainly comprising heart failure and ibrutinib. Terms pertaining to clinical treatment were brown and primarily evaluated for safety, biosimilar, and rituximab indicators. Terms correlated with adverse events were bright blue, involving biosimilars, public health, and capecitabine.

Burst analysis can be used to analyze research priorities and hot spots in specific periods. **Figure 8B** describes the burst analysis of the keywords between 2002 and 2021. Among the top 20 most explosive keywords, their strength ranged between 1.95 and 3.7. Their persistence ranged between 1 and 8 years.Their duration ranged from 1 to 8 years. From 2010 to 2018, the quality of life has occupied a long time, with the strengths of 2.21. This implies that improving the quality of life of cancer patients has always been a focus. Since 2018, immunotherapy, nivolumab, anti-CTLA-4, mechanism, and signal detection might be considered the frontier in pharmacovigilance and cancer research.

Thematic maps are primarily employed to characterize relationships between and within topics, and they can indicate the topic’s significance in the development of the field as a whole (31). The upper right cluster (Q1) reflects highly developed themes. The strong centrality and density usually represent the most advanced and vital topics in the research field and are related to many other topics. The upper left cluster (Q2) comprises low centrality and high-density clusters, highly developed but somewhat isolated. The lower left quadrant (Q3) represents emerging or descending topics, representing clusters with few or weak ties to other topics. The lower right quadrant (Q4) represents fundamental or horizontal themes, which means it has many relationships with other themes, but the relationships are weak. **Figure 8C** shows that the terms pharmacovigilance, adverse drug reactions and cancer are sandwiched between Q1 and Q4, indicating that the subjects were well-developed and can be used to construct research fields. Meanwhile, immune checkpoint inhibitors and immunotherapy sandwiched between Q3 and Q4 may indicate that it is the basic for future development of pharmacovigilance and cancer. Infliximab, multiple sclerosis (MS) and biosimilars in Q2 imply highly developed and isolated internal linkages, which may become the next hot spot for research if further links with pharmacovigilance and cancer are further strengthened. Among these, MS may be the type of disease that should be the focus of attention, as its treatment may increase the risk of cancer (30). Cancer treatment remains the primary target area among the clinical applications of biosimilars. More approved biosimilars are expected in the coming years, so comprehensive pharmacovigilance is necessary. It was reported that a patient with multiple sclerosis developed lymphoma eight months after exposure to teriflunomide (a drug for disease modification therapy) (32). Although it is the first published case of teriflunomide lymphoma, it indicates that there are specific cancer risk signals that need to be further evaluated. Infliximab, the first TNF-α antibody used to treat severe chronic inflammatory diseases, has some safety problems, such as infection or carcinogenesis, so a thorough evaluation of safety is helpful to its rational clinical use (33). The immunogenicity of biologics and the complexity of production make them more susceptible to various adverse reactions. Therefore, it is also important to strengthen the pharmacovigilance of biosimilars for safe clinical use (34, 35).

### Analysis of co-citation

**Figure 9:**
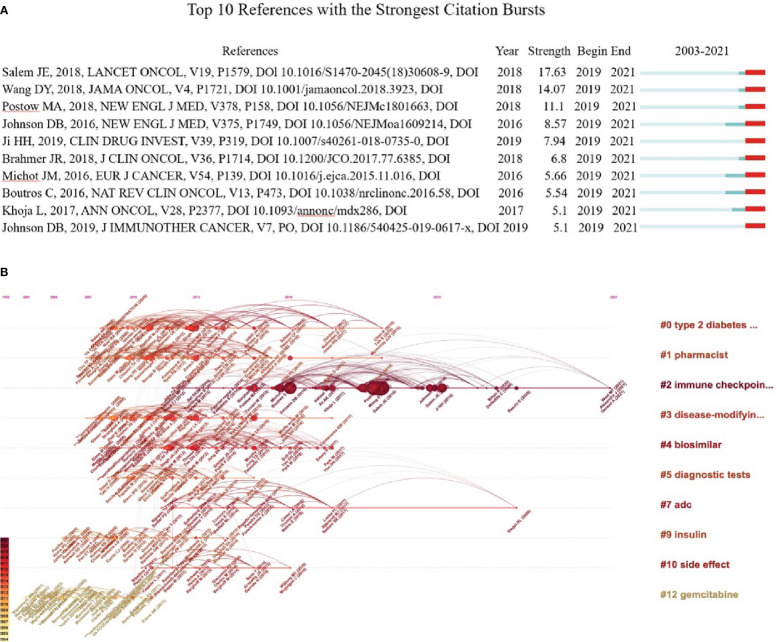
Co-citation analysis. **(A)** Analysis of the top 10 co-cited bursts. The blue line indicates the timeline from 2002 to 2021, and the red line indicates the point in time when each burst was co-cited. **(B)** Visual map of the timeline viewer associated with pharmacovigilance and cancer. The cluster tag sits at the far right end of each horizontal line. The node size on the line is proportional to the referenced quantity. The time at the top of the figure corresponds to the paper’s publication date. Nodes with darker labels reflect more recent literature, and nodes with more links indicate cocitations.

Co-citation refers that two or more papers being cited by one or more papers, which was used to evaluate the relevance between papers (36). The co-cited references were analyzed using CiteSpace. Both **Table 3** and **Figure 9A** show the top 10 highly co-cited references, each of which was co-cited over ten times. In 2018, an article authored by Salem JE., et al. on Lancet Oncol obtained the most co-citation (*n*=31) and a high burst (strength=17.63), lasting from 2019 to 2021. It illustrates that ICIs, as one of the primary means of cancer treatment, may lead to severe and fatal cardiovascular immune-related adverse events (CV-irAEs), including life-threatening myocarditis, pericardial disorders, and blindness associated with temporal arteritis, especially after drug combination (37). In addition, the assessment found that the relevant toxic effects caused by ICIs vary greatly between different treatment schemes, suggesting that individualized clinical treatment requires more rigorous planning and close monitoring (29).

**Table 3:** Top 10 co-citation in the field of pharmacovigilance and cancer.

Citation	Bursts	Title	Author	Year	Journal
31	17.63	Cardiovascular toxicities associated with immune checkpoint inhibitors: an observational, retrospective, pharmacovigilance study	Salem JE., et al	2018	Lancet Oncol
26	14.07	Fatal Toxic Effects Associated With Immune Checkpoint Inhibitors: A Systematic Review and Meta-analysis	Wang DY., et al	2018	JAMA Oncol
22	11.1	Immune-Related Adverse Events Associated with Immune Checkpoint Blockade	Postow MA., et al	2018	N Engl J Med
20	8.57	Fulminant Myocarditis with Combination Immune Checkpoint Blockade	Johnson DB., et al	2016	N Engl J Med
14	7.94	Adverse Event Profiles of Anti-CTLA-4 and Anti-PD-1 Monoclonal Antibodies Alone or in Combination: Analysis of Spontaneous Reports Submitted to FAERS	Ji HH., et al	2019	Clin Drug Investig
12	6.8	Management of Immune-Related Adverse Events in Patients Treated With Immune Checkpoint Inhibitor Therapy: American Society of Clinical Oncology Clinical Practice Guideline	Brahmer JR., et al	2018	J Clin Oncol
11	5.66	Immune-related adverse events with immune checkpoint blockade: a comprehensive review	Michot JM., et al	2016	Eur J Cancer
11	5.54	Safety profiles of anti-CTLA-4 and anti-PD-1 antibodies alone and in combination	Boutros C., et al	2016	Nat Rev Clin Oncol
10	5.1	Tumour-and class-specific patterns of immune-related adverse events of immune checkpoint inhibitors: a systematic review	Khoja L., et al	2017	Ann Oncol
10	5.1	Neurologic toxicity associated with immune checkpoint inhibitors: a pharmacovigilance study	Johnson DB., et al	2019	J Immunother Cancer

The LLR method was used to cluster the co-citation and visualization with a timeline view, which helps explore the research field’s evolution track and stage characteristics. In general, cluster modularity values (Q) > 0.3 indicate a significant cluster structuring, while average silhouette values (S) > 0.5 imply that the clustering is convincing (36). In this research, Q = 0.9529, and S = 0.9669, indicating significant clustering results. **Figure 9B** shows the top 10 clusters in the field of pharmacovigilance and cancer, which shows that the research hotspots of pharmacovigilance and cancer change over time. We can see that the ICIs (# 2) is the most prominent cluster with a long duration (2012–2021), indicating that it is a long-term research hotspot. Gemcitabine (# 12) and insulin (# 9) gained attention in the early period (2003–2006). Adc (# 7) has been concerned for a long time, from 2012 to 2020. Most research, such as pharmacist (# 1) and biosimilar (# 4), have emerged and continued since 2007 but disappeared after 2018. Thus, it is necessary to summarize the research in a timely fashion, which is the significance of the paper.

## Discussion

### General trends in pharmacovigilance and cancer research

There are 502 publications on pharmacovigilance and cancer, consisting of 378 articles and 124 reviews. According to the publications and citations from 2002 to 2021, pharmacovigilance and cancer research maintains a fluctuating upward trend. This change indicates scholars pay more and more attention to the pharmacovigilance and cancer, and predicts that more in-depth research will be published in the next few years. However, two points still need to be clarified. First, we tend to observe the overall research on drug safety, so pharmacovigilance rather than purely ADRs was chosen to study the relationship with cancer. Second, based on the first point, a total of 502 publications matching the topic were retrieved in this research, which is a small sample size compared to other articles of the same type, suggesting that related research on pharmacovigilance in oncology needs to be further improved.

The USA contributed most publications and citations. It may reflect the strong support of NIH and HHS, which provided financial assistance for over half of the publications sponsored.It may reflect the strong support of the NIH and HHS, which provided financial support for more than half of sponsored publications. In addition, most countries have direct and indirect collaborations with USA, which shows that USA plays a vital role in pharmacovigilance and cancer. This also shows that scientific research and economic development are inextricably linked. France is second to the USA regarding publications and citations. As the first country to create the concept of “pharmacovigilance”, France has maintained an active and cutting-edge research, making an outstanding contribution to the global pharmacovigilance (38). Also, European countries have close cooperation, which may be attributed to their geographical proximity. In addition, the links between other countries on the map, such as USA, China, France, Japan, and Spain, also indicate that international cooperation in pharmacovigilance and cancer research has gradually developed.

Identifying the most productive groups on a topic can be determined by analyzing collaborating institutions (39). Universities and research institutes are two leading institutions in pharmacovigilance and cancer, among which universities are still the mainstay of research. Vanderbilt University and Sorbonne University have the largest publications (*n*=22), indicating that these two institutions have made significantly contributed to pharmacovigilance and cancer. However, the contribution of other institutions to this area should not be ignored.

The co-authorship analysis identifies influential authors and potential collaborations. SALEM JE and LEBRUN-VIGNES B from Sorbonne University have published the most papers (*n*=20), and they have a solid cooperative relationship. The two researchers work together on the research of ICIs and cardiotoxicity studies with anticancer drugs (29). In addition, SALEM JE from Sorbonne University has the highest H-index (13), indicating his distinguished contribution in this field. SALEM JE and his colleagues found that treatment with ICIs can lead to serious and disabling inflammatory cardiovascular irAEs shortly after the commencement of therapy (37). Nineteen types of anticancer agents were found to be significantly correlated with atrial fibrillation (AF), which confirms that anticancer agents may be an independent risk factor for the progression to AF (28).

*Drug Safety* has the most publications and higher IF in this field, indicating that this journal has great academic influence. In addition, *Rheumatology* has the highest IF (7.046) but a small number of publications, suggesting that it is a potential platform for the future publication of pharmacovigilance and cancer research.

### Research hotpots of pharmacovigilance and cancer

From a bibliometric perspective, ICIs and Oncology biosimilars are cutting-edge research topics in pharmacovigilance that deserve our further attention.

### Pharmacovigilance for ICIs

ICIs have revolutionized cancer treatment (40). By blocking Immune checkpoint such as CTLA-4 or PD-1/PD-L1, ICIs can activate an immune response and effectively”release the brakes”on antitumor activity (41). Currently, FDA-approved ICIs include anti-PD-1 (eg, nivolumab and pembrolizumab), anti-PD-L1 (atezolizumab, avelumab, durvalumab), or anti-CTLA-4 (Ipilimumab), which have shown superior therapeutic effectiveness in non-small cell lung cancer, melanoma, lymphoma, renal cell carcinoma or other malignancies (42–45). However, abnormal activation of T cells also produces unique toxicities, termed immune-related adverse events (46). Moreover, the diversity of cancer types, the expanding indications for ICIs, and the combination of multiple drugs will lead to the emergence of more and more common and rare irAEs, which is a great challenge for pharmacovigilance.

The majority of irAEs are identified during pre-approval clinical development and primarily from spontaneous reporting systems and mandatory data reporting by pharmaceutical companies (i.e., post-marketing surveillance).Gastrointestinal and dermal toxicity are the most common irAEs (47). Several rare irAEs have been identified using ICIs, such as MS. MS is an immune-mediated inflammatory demyelinating disease of the central nervous system, and it is the most common cause of permanent disability in young people (48). Relapses of MS after ICIs are rare, but the adverse events described include rapid neurologic progression and death (49). Moreover, most patients continue to exhibit MS symptoms long after stopping therapy, which may be related to the long half-life of ICIs and the sustained immune response (50). Unexpectedly, some drugs approved for MS may have cancer risks, such as dimethyl fumarate, fingolimod, natalizumab and glatiramer acetate, which may be related to the occurrence of lung cancer, prostate cancer and breast cancer (51). It is still debated whether autoimmune disorders are contraindications to ICIs therapy, since some patients may still benefit from immune-based cancer treatments. In addition, ICIs have been detected for several emerging ADRs, such as ischemic heart disease and heart failure (52). Compared to other ICIs, nivolumab was the only one with a small increased reporting frequency of individual case safety reports with cardiac ADRs (53). Disproportionality analysis, causality assessment, and Drug Interaction Probability Scale algorithms are commonly used to analyze the collected data in pharmacovigilance studies to assess the relationship between ICIs and irAEs, the reported incidence of the phenomenon, and to provide a useful reference for effective risk management planning and clinical treatment planning. For example, disproportionality analysis was used to assess the association of ICIs with hematologic toxicity. The results showed that hematologic adverse reactions induced by ICI monotherapy (particularly anti-CTLA-4 therapy) were reported with high frequency and were exacerbated in multidrug combination therapy (54). This result reminds clinicians of the importance of early identification and management of ICIs-related hematologic irAEs. irAEs caused by ICIs are influenced by the type of cancer. Among patients with lung cancer and malignant melanoma, ICIs-treated had increased rates of cardiac events (arrhythmias, myocarditis or heart failure). The type of ICIs influences ICIs-induced adverse reactions. Ipilimumab is an anti-CTLA-4 agent with an irAE rate of 60–65%, with more than 40% of patients presenting with serious irAEs (grade 3–4) (55, 56). In contrast, irAEs caused by anti-PD-1 and anti-PD-L1 agents are less frequent and show a lower grade (57). Therefore, drug selection, early detection and diagnosis of irAE, and active management are urgently required.

Furthermore, pharmacovigilance research have shown that high-level irAEs are usually seen in combination therapy. Combination therapy of anti-PD-1 and anti-CTLA-4 increases the frequency and severity of irAEs. irAEs (grade 3–4) caused by combination of nivolumab and ipilimumab treatment was 59%, while the incidence of nivumab or ipilimumab alone was only 21% - 28% (58). Two melanoma patients who received epirubizumab and nivumab combined treatment developed fatal myocarditis, including myositis with rhabdomyolysis, early progressive and intractable ECG instability, etc (59). This means that routine cardiac testing, such as EGG or troponin levels, is necessary for early detection and prevention of adverse effects during routine ICIs therapy and trade-offs in dosing regimens.

Based on the urgent need for cancer treatment, the indications of ICIs are expanding, which requires pharmacovigilance as a “weapon” to protect the safety and effectiveness of drugs. At the same time, ICIs research for the treatment of malignancies has expanded to next-generation checkpoints such as LAG-3, TIM-3, TIGIT and VISTA, even though most are still in early clinical stages. For example, Relatlimab is expected to be the first LAG-3 antibody to market and become the third class of ICIs after CTLA-4 and PD-1/L1 (60). It also indicates that the focus of pharmacovigilance for this drug will soon shift to post-marketing, including individual case safety reporting and risk signal monitoring, thereby reducing and preventing the emergence of potential irAEs and safeguarding the safety of ICIs.

### Pharmacovigilance for TKIs

Tyrosine kinase inhibitors (TKIs) can effectively block tyrosine kinase activity and inhibit cell signaling, thus inhibiting tumor cell growth and proliferation. Over the past two decades, various robust and well-tolerated TKI with single or multiple targets has been developed, with significant targets including EGFR, VEGFR, PDGFR, KIT, BTK, ALK, ROS1, HER2, NTRK, RET, MET, MEK, and FGFR (61). TKIs have significantly improved survival and quality of life for patients with cancers such as lung, liver, gastrointestinal mesenchymal tumors, breast, and thyroid cancers, pushing tumor treatment from the era of chemotherapy to the period of targeted therapy with individualized and precise treatment. With the widespread use of TKIs, drug-related adverse reactions are also receiving increasing attention. A study analyzing the safety of TKIs for the treatment of non-small cell lung cancer through the Italian Pharmacovigilance database:not only confirmed the well-known risks of TKIs, which usually include skin, gastrointestinal, general, liver and respiratory diseases and infections, but also found the occurrence of life-threatening severe ADRs with TKIs, including respiratory failure, interstitial lung disease and cardiogenic shock, especially in young patients (62). A pharmacovigilance analysis of ALK-TKI inhibitors conducted through the FDA Adverse Event Reporting System (FAERS) identified several important safety signals, including pulmonary arterial hypertension, rectal perforation, myasthenia gravis, and photosensitivity (63). A disproportionality analysis of adverse events (AEs) for EGFR-TKI (gefitinib, erlotinib, afatinib, oseltinib) through FDA adverse event reports: EGFR-TKI-related AEs included skin, nails, gastrointestinal tract, liver, eyes, and lungs. Novel AEs were also identified, including “intestinal obstruction” and “hypokalemia” for gefitinib and erlotinib, and “hyponatremia” for gefitinib, erlotinib, and afatinib. “and “alopecia” with erlotinib (64). EGFR/ALK TKIs are associated with fatal interstitial pneumonia (IP), with erlotinib-induced IP having the highest morbidity and mortality rate (65). Use of VEGF-TKI increases risk of aortic coarctation (66). A pharmacovigilance survey based on the FAERS database found that the most common, high mortality adverse reactions to BTK inhibitors (ibrutinib and acatinib) were infections, such as pneumonia and pleural effusions, especially in elderly patients. In addition, cardiovascular-related adverse reactions, such as atrial fibrillation and heart failure, were fatal adverse events associated with ibrutinib (67). ALK and ROS1 inhibitors have a higher incidence of conduction disease and QT interval prolongation than other (EGFR, BRAF, MEK) targeted therapies. Compared to other EGFR inhibitors (erlotinib, gefitinib, afatinib), and targeted therapies, osimertinib is strongly associated with QT interval prolongation, SVT, and heart failure (68). Two cases of patients with advanced melanoma controlled by long-term MEK inhibitors or a combination of BRAF and MEK inhibitors who developed fractures associated with severe osteopenia (69).

In summary, the ADR of TKIs include gastrointestinal reactions, rash, edema and sodium retention, eye symptoms, oral mucositis, hypertension, proteinuria, malaise, hand-foot syndrome, liver injury, nail infection, interstitial lung disease, and cardiotoxicity. Therefore, clinical use should focus on monitoring TKIs causing serious ADR. For example, cardiotoxicity caused by ALK, ROS1, EGFR-TKIs; aortic coarctation caused by VEGF-TKI; pulmonary hypertension caused by ALK-TKI, interstitial pneumonia caused by EGFR/ALK TKIs, infection caused by BTK inhibitors and other adverse reactions.

### Pharmacovigilance for oncology biosimilars

Oncology biosimilars are a potential research hotspot. Biosimilars are close copies of patented biologics designed to cut prices to increase access for patients to affordable medications that are equivalent in safety and effectiveness to the originator drugs (70). In the biosimilars market, oncology biosimilars occupy the largest proportion, and their prices are at least 30% lower than those of the original drugs (71). Currently, many antitumor biosimilars such as trastuzumab-dkst biosimilars and bevacizumab-awwb have been approved for marketing, with outstanding effectiveness in treating malignancies such as metastatic breast cancer, non-small cell lung cancer, and cervical cancer (72, 73). However, there are many challenges to biosimilar implementation. In addition to the complexity of the development phase, the lack of extensive clinical data, the difficulty of market promotion and patient and physician acceptance, and ongoing pharmacovigilance are among the biggest difficulties facing generic drugs (74).

Immunogenicity testing has been a critical area for pharmacovigilance (75). Differences in formulation do not alter the effectiveness, originator and biosimilar products, but the risk of immunogenicity or intolerance may be increased (76). Therefore, it is important for the safe application of drugs to collect and evaluate the long-term safety data of marketed biosimilars. Extrapolation of indications (biosimilars can be approved for other indications of reference products without clinical trials) adds to the workload of pharmacovigilance of biosimilars. Furthermore, to ensure no additional safety concerns are present for a given indication with a biosimilar over the originator, characterisation of safety, immunogenicity and pharmacokinetic biodistribution must be present among the totality of evidence (77–79). All of the information that regulators must consider before making extrapolation decisions.

As more patents of antitumor biologics (such as daretozumab) gradually expire worldwide, the development pace of biosimilars in oncology will accelerate and expand. The powerful pharmacovigilance strategy ensures that any safety-related matters can be monitored, providing helpful information for the development and clinical application of these drugs.

### Challenges and opportunities of pharmacovigilance and cancer

Since the tragedy of thalidomide in the 1960s, pharmacovigilance has developed into an international superstructure that promotes the surveillance of drugs for human use (80). With the continuous improvement of the concept of pharmacovigilance, this work has already broken through the monitoring of adverse reactions after drug marketing, extended to the drug development phase and clinical research stage. Compared to other medical fields, the pharmacovigilance of oncology is not straightforward due to the intrinsic biologic toxicity of anti-neoplastic agents, narrow therapeutic windows, high doses and strict therapeutic schedule. Importantly, the continuous introduction of new types of drugs has also increased the complexity of pharmacovigilance. In the pre-marketing stage of anti-neoplastic drugs, the safety issues of drugs are mainly discovered through *in vitro* experiments, animal toxicology and clinical trials. However, these understandings and researches inevitably have limitations. The results of animal studies are insufficient to predict human applications’ safety. Clinical trials test drugs on relatively small samples of highly selected patients and monitor safety and outcomes for relatively short periods (81). Particularly, information on rare and severe adverse reactions, long-term toxicity, effects on special populations (e.g., children, the elderly or pregnant women), and drug interactions are usually incomplete in premarketing studies. Therefore, it is particularly important to carry out post-marketing surveillance of drugs. An essential challenge in this pharmacovigilance phase is collecting and assessing observational data on post-marketing drugs and drawing strong conclusions, which is a major component of ADR monitoring. Especially in oncology, there are more confounding factors. First, frequent use of multiple therapeutic regimens makes it difficult to determine whether the adverse reactions are caused by a single drug or an interaction between drugs and involves ‘innocent bystander’ effects (82, 83). Second, complex patient histories can lead to confounding and effect modification (i.e. drug-disease interactions). Then, the toxicity of anti-neoplastic drugs is generally considered “normal or unavoidable”, so the threshold for spontaneous ADR reporting is relatively high (82). In addition, post-marketing pharmacovigilance is largely based on spontaneous reporting, which implies a lack of data. In this case, the knowledge and attitude of health professionals are decisive factors in determining the quantity and quality of spontaneous reports (84). In the EU Directive, even a lack of effectiveness was considered an ADR because it may result from defective batches or errors in drug administration (85). Significantly, the lack of effective drug communication with patients is also one of the important reasons for ADRs (84).

Therefore, there is a need to strengthen pharmacovigilance in oncology in multiple ways, which is essential for the continued safety of the drug. Firstly, a complete set of risk management plans and pharmacovigilance measures should be developed early in drug development and accompany the drug throughout its life cycle. Secondly, the importance of pharmacovigilance education for healthcare professionals is emphasized to reduce the harmful phenomenon of underreporting. At the same time, cancer patients need detailed medication administration information, which can effectively improve patient compliance, reduce the occurrence of adverse events and ensure the effectiveness and safety of drug therapy. Thirdly, the focus of pharmacovigilance is on proactive detection. With the development of big data, artificial intelligence and other technologies, some emerging pharmacovigilance systems and analysis methods are gradually applied. The use of computer technology to diagnose and predict potential drug interactions and candidate biomarkers is essential for tailoring safety monitoring protocols and treatment decisions, especially in the early stages when there are no patient sample cohorts with adequate sample sizes (86, 87). The Sentinel initiative extends the pharmacovigilance capabilities by leveraging Electric Health Records systems and Insurance Claims data in distributed data networks of partner institutions, allowing for more comprehensive statistics on medication use and predicting the risk of drugs in advance (88). Similarly, Social media data often contain information critical to postmarket pharmacovigilance, such as individual experiences of ADRs, information about environmental factors and polypharmacy that is often missed by other postmarketing surveillance systems (89). In addition, the application of machine learning, natural language processing and cloud infrastructure allows for the rapid integration and analysis of data, significantly improving the efficiency of pharmacovigilance from all aspects.

In conclusion, the ultimate goal of pharmacovigilance is to ensure the safety of drugs and save lives.

## Conclusion

This research is the first using bibliometric analysis of pharmacovigilance and cancer linkages. Based on published academic literature, it provides a complete and exhaustive overview of trends and research fronts in pharmacovigilance and cancer. The USA, France and UK significantly contributed to pharmacovigilance and cancer research and were in a leading position in global research in this field. Salem JE and Lebrun-Vignes B of the Sorbonne University were two distinguished scientists who had a remarkable impact in this field. *Drug Safety* was significant in this field. The keywords and co-citation burst and clustering analysis showed that ICI has been a popular research topic and will continue to be in the future.This study can help researchers get familiar with the current situation and trend of pharmacovigilance and cancer research, and provide valuable reference for the selection of future research directions.

## Strengths and limitations

Although this study was a relatively objective and comprehensive bibliometric analysis and visualization of pharmacovigilance and cancer research, it still has limitations. First, only English language literature is included. For example, a large amount of Chinese literature was excluded, neglecting Chinese contributions to oncology-related pharmacovigilance. Second, the publications presented in this paper are limited to the WoSCC database. Researchers have widely embraced high-quality bibliometric analyses using the WoSCC database. Third, The continuous updating of the database leads to the change in bibliometric data, which may lead to different conclusions. Therefore, updated research work needs to track the latest primary and non-English studies.

## Author contributions

RS: Analyzed data and drafted original manuscript YX: Analyzed data and drafted original manuscript XP: Sort out data and relevant literature YW: Revised manuscript ZL: Revised manuscript XZ: Revised manuscript BZ: Provide main writing ideas, supervise and finalize the manuscript. All authors contributed to the article and approved the submitted version.
